# An Interesting Case of Abortion

**Published:** 1884-03

**Authors:** K. P. Moore

**Affiliations:** Macon, Georgia


### AN INTERESTING CASE OF ABORTI N.

BY K. P. MOORE, M.D., MACON, GA.

Was called, on night of 22d November, 1883, to see Mrs. P., age 35, mother of four children, youngest now little over two years. She was having a considerable uterine flow, with some periodical pains. She had been somewhat irregular in her catamenial periods, and the unusual flow and pains at this time would have caused her no special uneasiness but for the fact that she had passed a "fleshy mass," as she termed it. This "mass" was shown me, and certainly was placental tissue, though not a placenta in tact. She had passed two other pieces about the size of the thumb, which I did not see. I confidently told the husband and the patient that she had had an abortion, and that it was possible the fœtus had been taken away with the cloths and coagula which had been passing for a day or two previous to my visit. Or, I explained to them that the fœtus might yet be retained within the uterus, and in that event would likely pass during the night, or the next day. I ordered ergot, squibbs, fl. ext., in half drachm doses, to be repeated every two or three hours, or oftener, should the hemorrhage continue. I could not explore the uterus, hence I tamponed the vagina, and left her for the night. She had a moderately comfortable night; I found her in as good condition next morning as I could expect; there was no hemorrhage when I removed the tampon, and no fœtus. The cervix would not admit of an exploration, and as nothing seemed just then to demand an interference, I decided to leave her in the hands of nature. Directed the ergot to be continued, and dismissed her with instructions to keep very quiet; and should anything more pass from the uterus to send for me at once. I visited the store of the husband every day for some days, and learned that the patient was improving. She seemed to make a good recovery, and in a short while went to a neighboring city on ten days visit. Her health improved very fast while away, and she was looking quite restored on her return. She continued to improve, until, on the night of the twenty-fifth of December, she had gone with her family two or three blocks away to witness a display of fire-works, where she remained for two hours or more standing and sitting around on the damp, cold ground. She returned home about eleven o'clock, and retired, feeling quite well. She was awakened about twelve o'clock with a considerable hemorrhage. She at once resorted to the ergot, and by day it had ceased; so that she was up next day, and attended to her domestic duties. It being now about a month since her previous attack, she supposed it was a return of her monthly period, and hence felt no special concern. For several nights these hemorrhages would recur, only to cease by morning, and the patient would be up. In the meantime there commenced to pass pieces of offensive decomposed tissue, and other semi-organized lumps, which again caused anxiety, and I was sent for. I gave it as my opinion that these decomposed masses were remains of the old placenta, and upon seeing some of the vitalized tissue, I was satisfied that they were hydatidiform moles, and explained to the family that probably there were left remaining some villosities of the chorion, and that from them these vegetations had cropped out. Hence I suggested, as the rational plan of treatment, an exploration of the uterus, and mechanical removal of whatever might be there to produce this abnormal state of affairs, and this continued hemorrhage. Against this procedure my patient very strongly protested, and begged me to try all other means first, and let the proposed exploration and intra-uterine medication be used only as a *dernier resort.* This I consented to do under protest. For the next several days I went through the list of remedies for hemorrhage, both local and constitutional; but failed to correct the flow, which, while it was not very profuse, it was constant.

Finding my efforts had all failed, and my patient becoming exsanguinated and very weak, I urged intra-uterine interference. Late in the afternoon of January 10, 1884, I introduced through the cervical canal a sponge tent, and supported it with cotton packing. On the following morning, removed the packing, and found the tent saturated with blood; hemorrhage had gone on all night. The tent failed to dilate the canal sufficiently to introduce any instrument large enough to accomplish any good, hence I applied a still larger sponge tent, and left it in position until late in the afternoon, packing the vagina as before. On my evening visit, I found there had been no hemorrhage during the day, and I removed the cotton perfectly dry. The cervix was now tolerably well dilated, but the uterus was too high for me to reach the cavity with my finger, and the curette which I had with me was too large to be introduced; I therefore concluded to syringe out the uterus with solution tr. mur iron—one part to four; this I did with a long nozzle uterine rubber syringe. There being no hemorrhage, I packed the cervical canal and vagina with cotton, and left her till the following morning. At eleven o'clock next day, I reached my patient, and found her having considerable uterine contractions, which I attributed to presence of probable clot and the cervical packing; but, to my astonishment, when I examined, I found in the vagina, with the packing, a miniature though well-developed fœtus, which from its development, and the development of the placenta, must have been five or six months advanced. The placenta was well-organized, and as large as we would expect to find at five or six months gestation. It presented on one edge a ragged, disorganized appearance, and was rather corrugated and shriveled from the contact of the iron.

Now, the points of interest are these: Could there have been a real and false conception at the same time? And from whence came the placental tissue in the first attack on the 22d November?

If there had been twin conceptions in the outset, and if she really aborted one of them in her first attack, we could reasonably explain the hydatidiform growths, by supposing that some of the villi of the chorion remained and furnished a fruitful soil for their formation and growth. Could all this have gone on in the uterus, and the other fœtus continue to grow and develop?

The uterus was never large enough to be noticed or felt above the symphysis, and was not larger to the touch than we might expect from the inflammatory condition of the organ; and hence, this fact, together with the history of the case, precluded any suspicion of there being within the uterus a fœtus. Did the degenerative process going on militate so much against the growth of the fœtus as to account for its small size? If so, would it not also have interfered with its development as to form, etc.?

After the abortion on the 12th of January, the patient made a slow but good recovery; and at this writing, February 29th, is up, and in moderately fair health.

